# Selection of nitrogen responsive root architectural traits in spinach using machine learning and genetic correlations

**DOI:** 10.1038/s41598-021-87870-z

**Published:** 2021-05-05

**Authors:** Henry O. Awika, Amit K. Mishra, Haramrit Gill, James DiPiazza, Carlos A. Avila, Vijay Joshi

**Affiliations:** 1 Texas A&M AgriLife Research and Extension Center, Weslaco, TX 78596 USA; 2 Texas A&M AgriLife Research and Extension Center, Uvalde, TX 78801 USA; 3 Department of Horticultural Sciences, Texas A&M University, College Station, TX 77843 USA

**Keywords:** Environmental sciences, Plant sciences

## Abstract

The efficient acquisition and transport of nutrients by plants largely depend on the root architecture. Due to the absence of complex microbial network interactions and soil heterogeneity in a restricted soilless medium, the architecture of roots is a function of genetics defined by the soilless matrix and exogenously supplied nutrients such as nitrogen (N). The knowledge of root trait combinations that offer the optimal nitrogen use efficiency (NUE) is far from being conclusive. The objective of this study was to define the root trait(s) that best predicts and correlates with vegetative biomass under differed N treatments. We used eight image-derived root architectural traits of 202 diverse spinach lines grown in two N concentrations (high N, HN, and low N, LN) in randomized complete blocks design. Supervised random forest (RF) machine learning augmented by ranger hyperparameter grid search was used to predict the variable importance of the root traits. We also determined the broad-sense heritability (H) and genetic (*r*_*g*_) and phenotypic (*r*_*p*_) correlations between root traits and the vegetative biomass (shoot weight, SWt). Each root trait was assigned a predicted importance rank based on the trait’s contribution to the cumulative reduction in the mean square error (MSE) in the RF tree regression models for SWt. The root traits were further prioritized for potential selection based on the *r*_*g*_ and SWt correlated response (CR). The predicted importance of the eight root traits showed that the number of root tips (Tips) and root length (RLength) under HN and crossings (Xsings) and root average diameter (RAvdiam) under LN were the most relevant. SWt had a highly antagonistic *r*_*g*_ (− 0.83) to RAvdiam, but a high predicted indirect selection efficiency (− 112.8%) with RAvdiam under LN; RAvdiam showed no significant *rg* or *rp* to SWt under HN. In limited N availability, we suggest that selecting against larger RAvdiam as a secondary trait might improve biomass and, hence, NUE with no apparent yield penalty under HN.

## Introduction

Being the first tissues that intercept various nutrients and water uptake, roots play an essential role in plant growth and development. Root architecture highly varies in response to different nutrient deficiencies^1^ and adapts to continually changing growth conditions through structural plasticity^2^. Most plants can utilize only half of the applied N, losing it in the form of nitrates (NO_3_^−^), which can cause environmental hazards^3^. Spinach requires high rates of N fertilizer to produce high biomass and quality. It is estimated that about 60% of N applied to spinach in commercial production is lost through leaching^4^ due to its shallow root system^5^ and short production cycle. Spinach is also relatively poor in its NO_3_^−^ reducing capacity^6,7^. About 80% of the total root length of spinach settles in the upper 0–15 cm soil layer, and the root distribution is not affected even by the addition of usable nitrates below 15–30 cm^5^. Thus, the rooting structure and the growth patterns that define roots are essential considerations to delineate the differences in nutrient absorption and efficiency. This is even more important in spinach due to its high affinity for N^8^. The morphological changes in the root system are regulated by the plant's nutritional status and interaction with the surrounding environment as detected through the localized signals by roots. Several studies have discussed the N-dependent (NO_3_^−^, ammonium and glutamate) changes in the root architecture across species^9–13^. Therefore, investigation of root development is of importance for understanding plant responses to low-N stresses.

Unlike soil, where the complex root-microbial association may naturally facilitate the plant absorptive capacity of hitherto unavailable N^14,15^, root development in the soilless media is entirely reliant on an exogenous supply of nutrients. Due to their inert nature, the soilless systems minimize the changes that could occur due to gradients in temperature, oxygen status, water availability, pH, bulk density, or seasonal variations. In these cases, the root structure and appearance are a function of genetics as modified by the soilless matrix, and the concentration of the nutrients applied^16^. Different studies have profiled root systems and their association with the environment in field conditions, non-soil media and microbes^17–19^. Modeling techniques for root feature diversity, structure, and activity have been attempted, including multivariate and machine-learning techniques^20–22^. However, much remains to decipher the genetic and potential importance prioritization of the assigned root traits in influencing the above-ground biomass.

Root structure and its functioning are associated with N uptake, which influences plant performance and yield. Although improving root performance is relevant to all crops, it is particularly relevant to short-cycle vegetable crops like spinach that would benefit from early below-ground vigor^23^. However, the shorter crop duration of spinach allows only a short time for root development^24,25^. To date, much research on developing breeding strategies to improve N uptake or utilization is focused on modifying the root architecture of main crops like maize, rice, and wheat. Although several root architectural, topological, and developmental traits such as deeper and longer roots, rapid growth, and higher root density associated with higher N use efficiency have been identified^26,27^ in cereal crops, such efforts to identify root traits that respond to N stress to capture the N available at depths in vegetables are limited.

Soilless indoor farming is becoming increasingly popular in the recent years^28,29^. Since a soilless indoor system relies on the artificial supply of essential nutrients^28–30^, nutrient management is critical for the harvestable quality and quantity of a crop^31,32^. The differences in the value for money' between varieties of the same species may sometimes narrow down how efficiently the crop can uptake and convert the nutrients into harvestable products. Rooting system and architecture are important determinants of efficiency that maintain a favorable balance between resource investment (photosynthates) and resource acquisition (raw materials)^33,34^. Varieties with favorable root architecture that enhance nutrient uptake and photosynthate use will reduce operating costs while balancing nutritional content and yield. This study investigates root traits and their relationship to the harvestable 'above-ground' biomass of spinach grown in two contrasting N supplies in a uniformly controlled indoor environment. Our assumption here is that in a uniform soilless matrix, genetics and the N management are the two primary sources of variation defining root architecture. We have used supervised random forest optimized machine learning algorithm by 'ranger'^35^, to predict the importance of eight root traits on the spinach's harvestable biomass at the 'baby-spinach' stage. We have also applied META-R^36^ to determine the genetic and phenotypic correlations and heritability to prioritize the root traits in influencing the harvestable shoot biomass. Finally, we compare the machine learning classification and the prioritization based on genetic correlations and present the top trait(s) with the highest selection potential.

## Methods

### Plants, plant material, experimental setup, and evaluation environment

A collection of 202 spinach (*S. oleracea*) accessions maintained and provided by the USDA-National Plant Germplasm System (NPGS) (https://npgsweb.ars-grin.gov/) at Ames, Iowa, U.S.A was used in the present study. The plants were grown in a growth chamber under controlled conditions of 12/12 h (light/dark), 22 °C, and 75% relative humidity. The seeds of each accession were sown in triplicate in turface (Turface Athletics MVP, PROFILE Products LLC, Buffalo Grove, Illinois, USA) in small pots (10.2 cm × 10.2 cm and 8.9 cm deep). Each set of replicates was completely randomized across separate shelves. After the seedling emergence, plants were fertilized with Peters professional ready mix (5-11-26, hydroponics special water-soluble fertilizer, Everris NA Inc., Ohio, USA) every after four days. Two concentrations of nitrogen (N), low N (50 ppm), and high N (200 ppm) were used for low and high N management, respectively. An additional N for the high N-management was provided using calcium nitrate, and equivalent calcium (3.85 mM) was replaced by calcium chloride in the low N-management. The concentrations of the macro/micro-nutrients present in the fertilizer solution are provided in Supplementary Dataset Table S1.

The experimental research in the lab facilities for this study was performed as required by Texas A&M System Regulation (15.99.06 Use of Biohazards in Research, Teaching, and Testing) and the University’s Rule for the use of Biohazards and Dual Use Research of Concern (15.99.06.M1 Use of Biohazards, toxins and rDNA and DURC), approved by the Texas A&M Institutional Biosafety Committee (IBC).

### Plant material processing, root imaging, and data processing

The plants were harvested at the physiological maturity of baby spinach (5–6 leaves) after 41 days of sowing. Each plant was carefully pulled from the turface and washed with running water to clear any debris off the roots. The roots were separated from the shoot at the cotyledonary nodes and floated in water. The lateral roots were separated gently using a fine tip paintbrush to minimize the overlapping of roots. The images were taken by digitally scanning roots of individual plants (Supplementary Dataset Figure S1) using a high-resolution scanner (Calibrated Color Optical scanner STD4800 with Special Lighting System) and scanned images analyzed using WinRHIZO Pro software (Regent Instruments Inc. Canada). Categorization of the traits was adapted from the Fine-Root Ecology Database^37^ classification and included: (1) morphology for root length (RLength) and average root diameter (RAvdiam); (2) an indication of the complexity of the root system architecture measured using number of tips, forks (number of root bifurcations), and crossings (Xsings; overlapping parts); (3) root system of the standing crop (RSSC) for root volume (RVol), root surface area (RSarea) and root weight (RWt). The harvestable above-ground biomass was determined as fresh shoot weight (SWt) (WR P-series balance, Model 500P, VWR International, U.S.A.) after removing the excess surface moisture by gently paper-bloating the wet roots followed by a two-minute air drying.

### Data analysis

The analysis pipeline was designed to define the phenotypic, genotypic, and predictive relationship between the root traits and between the root traits and the SWt of spinach plants grown in a soilless system. We determined the *r*_*g*_ and *r*_*p*_ (defined below in the section -Determining the genotypic and phenotypic correlation between traits) between root traits and *r*_*g*_ and *r*_*p*_ between the root traits and the SWt within and between the two N managements. Parallel to the correlation analyses, we used a supervised random forest machine learning^38,39^ technique to estimate the variable importance of each root phenotype in predicting the above-ground shoot biomass. The details of the parallel procedures are described below.

### Individual trait and combined management variance analysis and mean separation

Linear mixed models were implemented in *lmer* from package *lme4* of R using REML via Multi Environment Trial Analysis with R, META-R^36^, to calculate the adjusted means (best linear unbiased estimates, BLUEs, and predictors, BLUPs) for each root and shoot variable, under the two N managements. For individual analyses, we used the model is

1 $$Y_{ik} = \mu + Rep_{i} + Gen_{k} + \varepsilon ik$$

where Y_ijk_ is the trait of interest, *µ* is the mean effect, *Rep*_*i*_ is the effect of the* i*th replicate represented by the complete blocks, *Gen*_*k*_ is the effect of the *k*th genotype, *εijk* is the error associated with the *i*th replication, *j*th incomplete block and the *k*th genotype, which is assumed to be normally and independently distributed, with mean zero and homoscedastic variance σ^2^. For the combined analyses across the two N-managements, the model was adjusted to

2 $$Y_{ijkl} = \mu + Mgt_{i} + Rep_{j} \left( {Mgt_{i} } \right) + Gen_{l} + Mgt_{i} \times Gen_{l} + \varepsilon ijkl,$$

where the new terms *Mgt*_*i*_ and *Mgt*_*i*_ × *Gen*_*l*_ are the effects of the ith N-management and the N-management by genotype interaction, respectively. Genotype and N-management were both treated as random effects, and BLUPs were used to estimate random effects and BLUEs to estimate the fixed effect. Grand means were separated based on Fisher’s Least Significance Difference (LSD) at α = 5%. We also determined the coefficients of variation (CV) for all traits.

### Heritability

We estimated the average broad-sense heritability (repeatability, *H*) of three replicates in each N-management, which is also an estimate of correlation expected between line means from the three replicate trials conducted in the two N-managements. *H* was determined^40^ on a line mean basis as

3 $$H = \sigma^{2}_{g} \times \sigma^{2}_{g} + \sigma^{2}_{e} /nReps,$$

and combined for the two N-managements as

4 $$H = \sigma^{2}_{g} \times \sigma^{2}_{g} + \sigma^{2} ge/nMgt + \sigma^{2}_{e} /\left( {nMgt \times nReps} \right),$$

where *σ*^2^_*g*_ and *σ*^2^_*e*_ are the genotype and the residual error variance components, respectively; *nReps* is the number of replicates, *σ*^2^_*ge*_ is the variance component of genotype by N-management interaction, and *nMgt* is the number of N-managements in the analysis.

### Determining the genotypic and phenotypic correlation between traits

Genetic and phenotypic correlations were calculated for each trait pair, within and across the N-managements. The *r*_*g*_ were also determined in META-R, which applies the equations from Cooper et al.^41^. Between the N-managements, *r*_*g*_ was estimated as

5 $$r_{g} \left( {jj_{0} } \right) = r_{p} \left( {jj_{0} } \right)/H_{j} H_{j0} ,$$

and between traits within a single N-management,

6 $$r_{g} = \sigma_{g} \left( {jj_{0} } \right)/\sigma_{g} \left( j \right)\sigma_{g} \left( {j_{0} } \right),$$

where *r*_*p*_(*jj*_0_) is the phenotypic correlation between N-managements *j* and *j*_0_; and *H*_*j*_ and *Hj*_0_ are the heritability of N-managements *j* and *j*_0_ respectively, *σ*_*g*_(*jj*_0_) is the arithmetic mean of all pairwise genotypic covariances between trait *j* and *j*_0_, and *σ*_*g*_(*j*)*σ*_*g*_(*j*_0_) is the arithmetic average of all pairwise geometric means among the genotypic variance components of the traits.

For graphical illustrations, cluster analysis based on the environment distance matrix (1—Genetic Correlation matrix) was also performed using the ‘Ward’ method^42^, creating a dendrogram. In each case, a minimum heritability threshold was set at 0.1; any trait whose heritability within or between the two N-managements was lower than 0.1 was excluded from the analysis and was not plotted. For phenotypic correlations, simple Pearson correlations between different pairs of N-managements or traits were used.

### Predicting correlated response

Correlated response (CR) was predicted for SWt to determine if direct or indirect selection resulting from selecting a root trait would be superior under similar N-managements. We used the formula:

7 $$CR_{swt} = ir_{g} \surd H_{RT} V_{g(swt)} ,$$

where *CR*_*swt*_ is the correlated response of *SWt*, *r*_*g*_ is the genetic correlation, *H*_*RT*_ is the repeatability of root traits, *V*_*g*(*swt*)_ is the genetic variance of *SWt*, and *i* is the selection intensity whose estimate we assumed would be similar between traits. Thus, *CR*_*swt*_ was compared to direct response (R),

8 $$R_{swt} = i\surd H_{swt} V_{g(swt)} ,$$

by *CR*_*swt*_/*R*_*swt*_ = *r*_*g*_*√H*_*RT*_/*√ H*_*swt*._ That is, if r_g_ × H_RT_ > H_swt_, then indirect selection would be superior^43–46^.

## Summary of data preparation and evaluation by machine learning

To rank the root traits by importance in the prediction models for SWt, we used the random forest (RF) modeling in R. RF is a powerful ensemble machine learning tool that combines the outputs of numerous decision tree classification models. We applied the regression type to the *randomForest*^47^ package and first ran the regression on default tuning parameters. We also invoked a user-defined hyperparameter tuning in the *ranger*^35^ package to optimize our models; *ranger* is a C++ implementation of Breiman's FORTRAN-based random forest algorithm^39^. Finally, we compared the accuracy of the model from the *randomForest* default tuning and that from *ranger* hyperparametric search tuning. The function *missForest*^48^ was used to impute missing data. Outliers were normalized by an internally derived proximity matrix procedure built into the RF. In the normalization, if an outlier case *i* and case *j* both end up in the same tree node, increase proximity prox(*ij*) between *i* and *j* by 1 and accumulate over all trees in RF, the outliers are normalized by twice the number of trees in RF. This creates a proximity square matrix where observations that are ‘similar or alike’ in value have proximities close to 1 and the dissimilar proximity closer to 0.

### Default tuning and model evaluation

The default data split (into 63.25% as training dataset and the remainder as the validation set) were applied to train each N-management. The 63.25% is the proportion expected of unique observations in a bootstrap sample^39,49^. The typical range is ~ 60 to 85%, where smaller sample sizes can reduce the training time but may introduce more bias than necessary, while too large a sample size can increase performance but at the risk of overfitting because it introduces more variance^39^. An F-fold cross-validation feature in RF invokes the evaluation of model performance by training it on a number of different smaller datasets and evaluating them over the other smaller testing sets. By default, *randomForest* randomly splits the number of datasets of almost the same sized k-folds, and each of the folder models is evaluated over the number of folders and tested on the remaining test set^39,47^. This process is repeated until all the subsets have been evaluated. The regression tree parameters are tuned further by choosing the number of independent variables (m) using the default as *m* = *p*/3, where *p* is the total number of root traits in our analysis. This helps generalize the data best to return the least out-of-bag (OOB) error rate and provides a built-in validation set. Further, it identifies the number of trees (*ntree*), required to stabilize the error rate during tuning more efficiently^39,49^. OOB error is an internal error estimate of a random forest as it is being constructed^39^. It is estimated by testing each tree built from the bootstrap aggregation (bagging samples) from the training set on the remaining (validation set as defined by the default data split) of the samples not used in building that tree; randomForest chooses a random subset of features and builds many regression trees, and the model averages out all the predictions of the decision trees.

### Setting hyperparametric tuning and evaluation parameters

**Figure 1:**
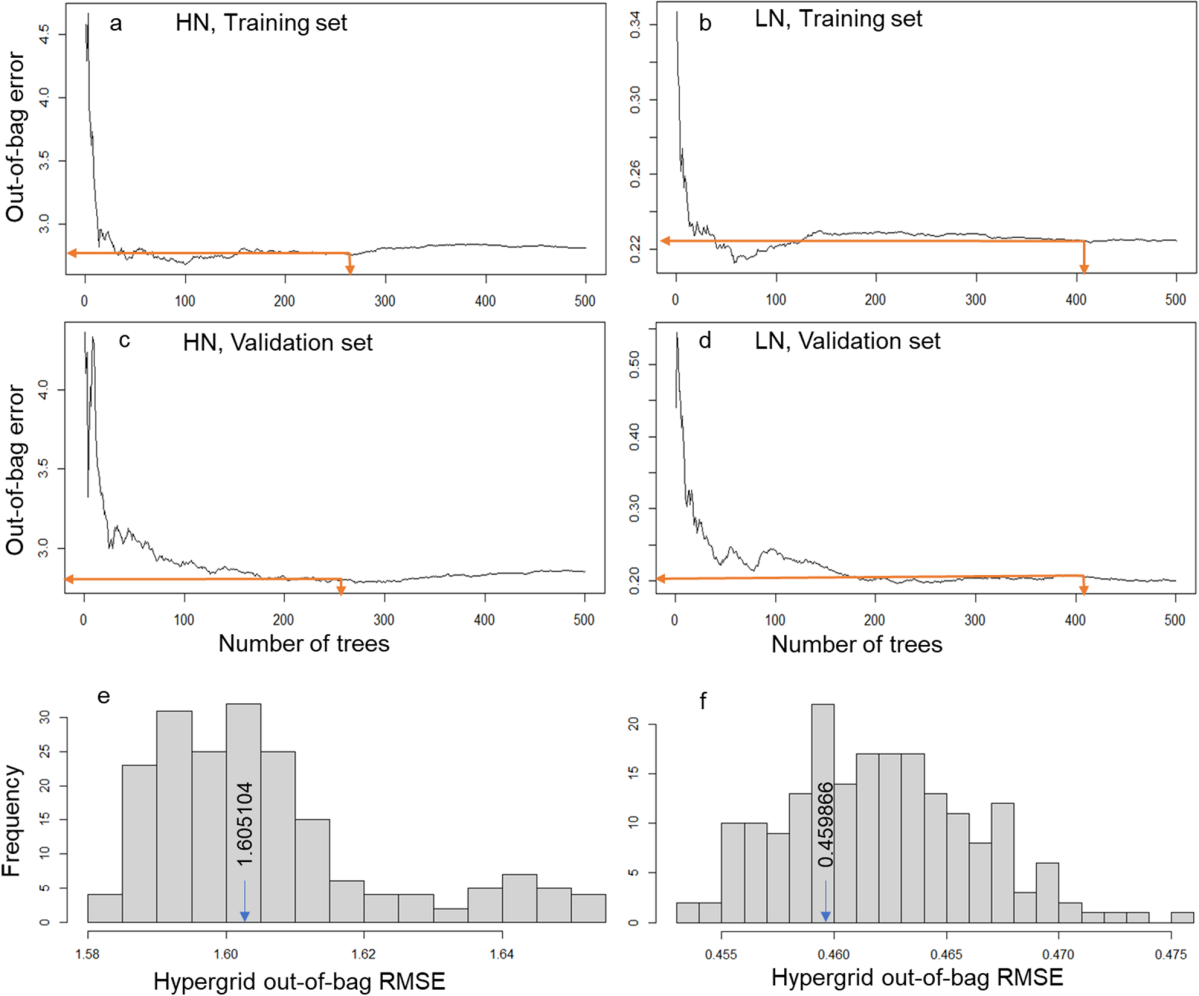
Model training and grid search across 192 hyperparametric combinations. Random forest out-of-bag error rate (**a** and **b**) compared to the corresponding validation error rate (**c** and **d**) averaged along tree optimization splits. The optimal number of trees and the corresponding mean squared error (out-of-bag error) are shown by the orange arrows on the graphs (**a**–**d**). The root mean squared error (RMSE) at which all hyperparameters converged is shown in the histograms (**e** and **f**). RMSE at optimal hyperparameters (obtained for training model and used for independent validation sets) are shown by arrows in the histograms Also see Supplementary Table S2-tuning hyperparameters).

We first determined the optimal number of trees (*ntree*), producing the least OOB error rate. The term ‘Optimal’ refers to the number of trees that were just enough to stabilize the OOB error and improve efficiency by avoiding unnecessary runs, as determined from the *ntree* function and *which.min* argument. The optimal number of trees was delineated first by running 500 trees with the default 63.25:36.75 split for each N-management. A hypergrid search was then constructed across several hyperparameter combinations and looped through each combination (details are in Supplementary Dataset Table S2). The model was evaluated over all the combinations we passed in the search space function using the grid search. The hyperparameter searches applied (values in parenthesis) were: *mtry* (4 variables from 2 to 48), for the number of random root trait variables to include in each tree. The primary concern was to tune the number of candidate variables (features) to sample at each tree node split randomly; 2) *sampsize* (sample fractions 0.55, 0.60, 0.65, 0.70, 0.75, 0.80) denoting the number of samples to train, 3) model *nodesize* (8 variables from 1 to 48), which determines the minimum number of samples within the terminal nodes and thus controls the complexity of the trees. This was necessary to set a bias-variance tradeoff where smaller node size allows for deeper, more complex trees with the risk of introducing more variance (risk of overfitting) and larger node results in shallower trees which may introduce more bias (risk of not fully capturing unique patterns and relationships in the data)^39^. The minimum OOB root mean square error (OOB_RMSE) was set at zero (0). For *ntree*, we used 500 because the OOB_RMSE from hypergrid searches stabilized with less than 500 trees (Fig. 1). The resulting hyperparameter combination producing a model with the least prediction variance and OOB_RMSE was selected and tested with the training set and an independent, smaller test sample data (not used in each N-management training). The independent test sample was obtained from the optimal *sampsize* split, without bootstrap replacement.

### Constructing accuracy function and evaluating the models

We applied all the above models on the same independent test (validation set) dataset to evaluate the accuracy of the grid-tuned model compared to the default model. The best of the two (lower mean error rate and greater mean model R^2^ of regression trees) was used as our prediction model in a new regression run in *randomForest* to predict the new test set. The validation set was used as the independent test set since the *sampsize* split was done before bootstrapping and before *sampsize* split-variable randomization of the predictor root traits. Furthermore, we set importance as equals impurity' in the above modeling, which allows us to assess the variable importance of the root traits. Variable importance is measured by recording the decrease in mean square error (MSE) each time a variable is used as a node split in a tree^39^. The remaining error left in predictive accuracy after a node split is the ‘node impurity,’ and a variable that reduces this impurity is considered more important than those that do not. Consequently, the root variable with the greatest accumulated reduction in MSE was considered the more impactful^39^.

## Results

### Model tuning and accuracy

Optimized hyperparameters used in cross-validation with both the training and test samples in the two N-management datasets were variable size, node size, sample size, and the number of trees. All the optimized settings resulting from hyperparameter grid search are in Table 1, and the stabilizing *ntree* and OOB-RMSE are shown by arrows in Fig. 1a–f, respectively. For each N-management, hyperparameters were constructed (tested) across a total of 196 models (combinations: 8 predictor parameters [all the eight root traits], 4 node sizes [2, 4, 6, 8], across 6 sample sizes [0.56, 0.60, 0.632, 0.70, 0.74, 0.80], and 1 predetermined optimal number of trees [within 500], Supplementary Table S2). To assess the performance of our tuned hyperparameters, we compared the mean OOB prediction error, and the mean OOB variance explained (R squared_OOB, Table 2) between the tuned OOB regression model (training) and the test model, and between the training model and the RF default models. The OOB prediction error of 0.210 g of SWt under LN and 2.712 g of SWt under HN for the trained model (Table 1) were marginally smaller (the smaller, the better) than those for the RF default cross-validation (LN, 0.227 g; HN, 2.794 g) and the test model (LN,0.239 g, and HN, 2.799 g). Here, we define a marginal' difference as separation by at least 1%, but not large enough to be statistically significant by the conventional (non-machine learning) mean separation methods. The prediction variance explained (R squared_OOB) by our tuned model (57.2% of SWt) was similar to that of the internally cross-validated (default RF) model (56.8% of SWt) under the LN management but marginally larger (61.3% of SWt) than the default (60.2% of SWt) under the HN management. The hyperparametric tuning performed marginally better in the test model, with 61.2% and 64.6% variance predicted for SWt under the LN and HN managements, respectively. Overall, the tuned model performed as expected (with no large penalty even with varying sample size) on the independent test data.

**Table 1:** Summary of model evaluation and test (validation) by random forest machine learning.

N-management	Model name	Tuning RMSE at optimal hyperparameters	Optimal model parameters					Plit rule	OOB prediction error (MSE, mean of squared residuals)	Regression R-squared (OOB)
			Trees^a^	Nodes	Sample fraction^φ^	Variables^b^	Sample size			
LN	Default	0.4530081	500	5	0.632	2	127	Variance	0.227015	0.568
	Training		500	8	0.7	3	141	Variance	0.239117	0.572
	Validation		500	8	0.7	3	61	Variance	0.209563	0.612
HN	Default	1.581197	500	5	0.632	2	121	Variance	2.793847	0.602
	Training		500	2	0.56	7	121	Variance	2.798641	0.613
	Validation		500	2	0.56	7	81	Variance	2.712218	0.646

^a^Trees maintained at 500 since all optimal trees (in Fig. 2) fell within this limit.

^b^Number of predictor root traits sampled at each tree node; HN, high N; LN, low N.

^ϕ^The validation sample was kept independent of the training sample, and the same hyperparameters were applied as for the training model.

**Table 2:** The genetic correlations and phenotypic correlations of root traits.

Traits	SWt	RWt	RLenght	RSarea	RAvdiam	RVol	Tips	Forks	Xsings
SWt		0.789	0.766	0.759	0.168 ns	0.732	0.794	0.709	0.702
RWt	0.804		0.921	0.950	0.348	0.955	0.869	0.886	0.856
RLenght	0.769	0.952		0.987	0.144 ns	0.951	0.947	0.968	0.972
RSarea	0.757	0.967	0.992		0.282	0.988	0.915	0.953	0.939
RAvdiam	0.045 ns	0.199 ns	0.010 ns	0.135 ns		0.402	0.042 ns	0.102 ns	0.021 ns
RVol	0.730	0.964	0.968	0.992	0.254		0.864	0.916	0.887
Tips	0.815	0.923	0.968	0.936	− 0.112 ns	0.890		0.930	0.931
Forks	0.703	0.921	0.989	0.973	− 0.053 ns	0.941	0.958		0.988
Xsings	0.706	0.902	0.988	0.965	− 0.107 ns	0.927	0.966	0.995	

The table shows the genetic correlations (lower triangle) and phenotypic correlations (upper triangle) of eight root trait and one shoot trait under high N-management.

SWt, shoot weight, RWt, root weight, RLength, root length, RSarea, root surface area, RAVdiam, root average diameter, RVol, root volume, Tips, number of root tips, Forks, number of forks, Xsings, number of crossings, ns, not significant at P ≤ 0.05; all others, significant at 0.05 > p < 0.001.

### Prediction by machine learning is a close approximation of both the genetic and phenotypic correlations

**Figure 2:**
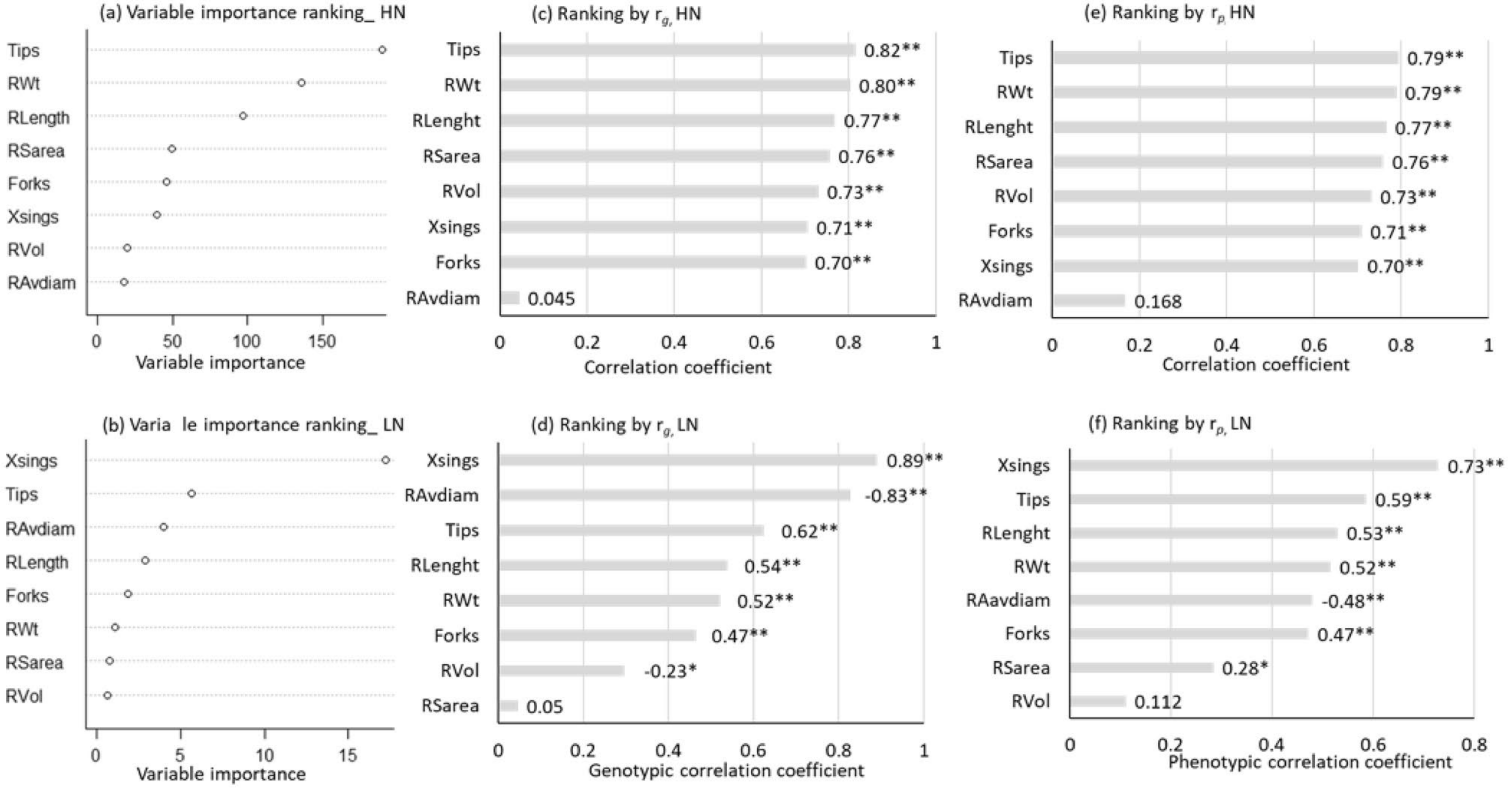
Ranking of root traits based on machine learning and multi-environment trait analysis methods. (**a**) and (**b**) are rankings of the variable importance of eight root traits (vertical axis) in a model for predicting shoot weight under high N-management, HN (**a**) and low N-management, LN (b) using random forest machine learning algorithm. Each point in (**a**) and (**b**) is a cumulative reduction in mean square error (MSE) of regression models each time the corresponding predictor root trait variable is used as a node split in a decision tree. The larger the cumulative reduction in the MSE, the greater the important ace and the higher a root trait ranks across all models tested. The traits have been ranked in ascending order with the least important at the bottom and the most important at the top on the vertical axis. (**c**) and (**d**) show the genetic correlation coefficients (*r*_*g*_), and (**e**) and (**f**) the phenotypic correlation coefficient (*r*_*p*_) between each variable and shoot weight. Correlation size and significance are shown on each bar. A negative ( −) before a number indicates it is a negative correlation, *significant at P = 0.05 and **significant at p < 0.01.

By machine learning (ML), we ranked root traits based on the predicted importance of each in the models in describing its relationship with SWt. The traits with the greatest variable importance (Tips under HN and Xsings under LN) identified by ML also had the largest *r*_*g*_ and *r*_*p*_ to SWt in the corresponding N-managements (Fig. 2). The traits with the least variable importance (RAvdiam under HN and RVol under LN) were correctly identified in three out of four cases by the *r*_*g*_ and *r*_*p*_ ranking methods; the exception was *r*_*g*_ in the LN, where RVol followed RSarea as the least correlated to SWt. Overall, 6 out of 8 traits were correctly matched between ML and *r*_*g*__HN, with a two-trait *r*_*g*_ position switch, e.g., RVol then Xsings, instead of Xsings then RVol. Only 2 out of 8 traits were correctly matched between ML and *r*_*g*__LN with a two-trait position switch between six traits, e.g., RAvdiam then Tips, RWt then Forks and RVol then RSrea, instead of vice versa (Fig. 2). These two-trait switches, in our opinion, are minor alterations if we consider the fact that the four root traits predicted by ML as the most important and the four predicted as the least important under LN were also the same root traits with the largest and smallest *r*_*g*_ and *r*_*p*_ under LN. The four traits predicted by ML as the most important and the least important under HN were the same for *r*_*p*__LN except for RWt instead of RAvdiam for *r*_*p*__LN. It seems that as the *r*_*g*_ and *r*_*p*_ decrease so does the ability of our ML variable importance predictions to correctly identify the ranking of root trait-SWt genotypic and phenotypic correlations, and vice versa.

### Pairwise genetic and phenotypic correlations are affected by N management

**Figure 3:**
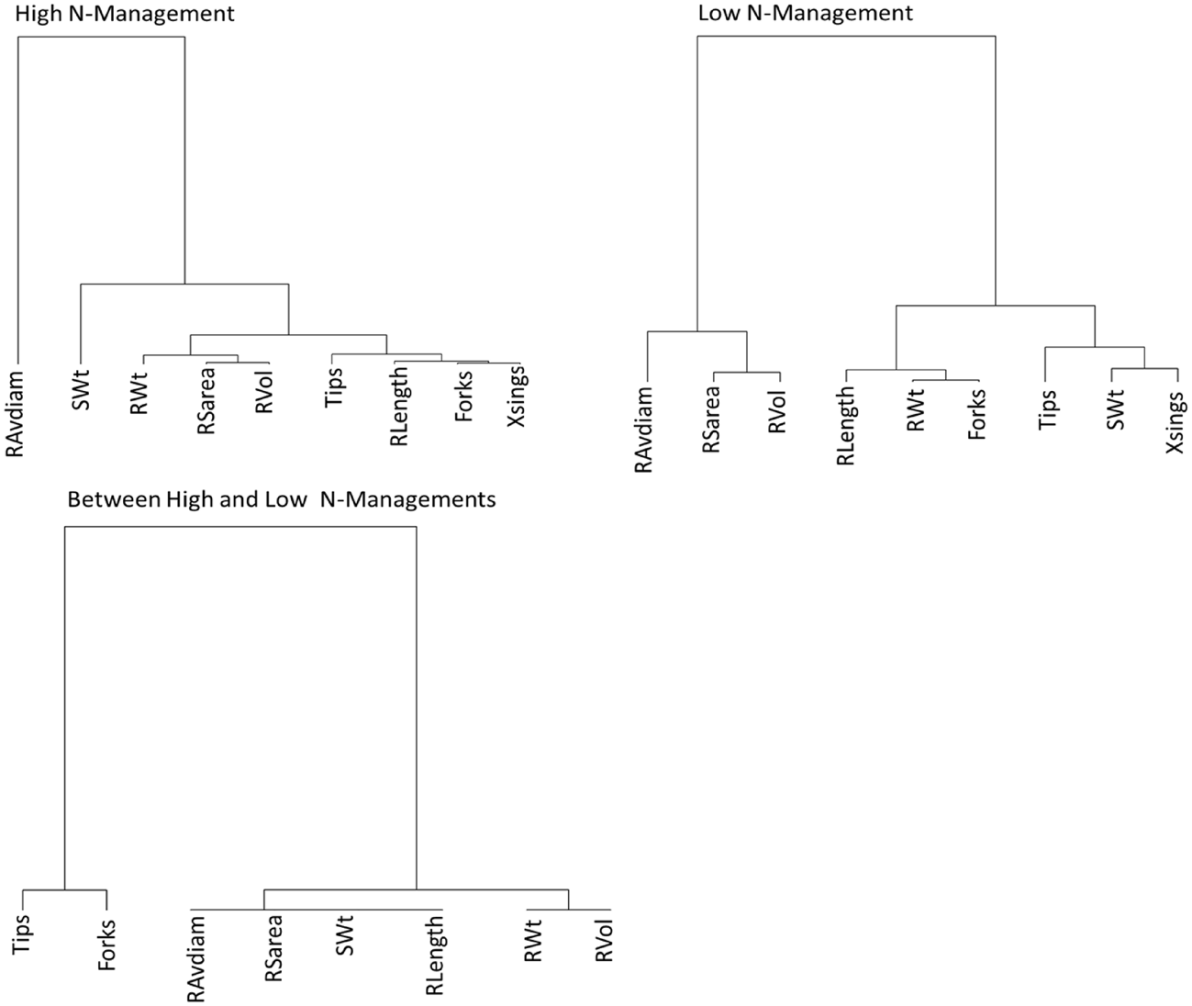
The structure of genetic correlations between traits within and between the two N-managements. Ward method was used to construct the dendrograms. In the correlations between N-Managements, Xsings is missing because its heritability was < 0.1 threshold we had set when calculating the genetic correlation coefficients.

The *r*_*g*_ between all traits within and between N-managements are summarized in Tables 2, 3 and 4, while the structure of these correlations is shown in Fig. 3. The main reason for estimating *r*_*g*_ is to determine if a greater response on SWt would result by selecting a root trait as a secondary trait. The pairwise *r*_*g*_ and *r*_*p*_ between root traits and SWt were nearly identical under HN except for correlations between RAvdiam andSWt where *r*_g_ = 0.045 and *r*_*p*_ = 0.168. Under LN, the similarity was also high except for correlations between RAvdiam and SWt where *r*_*g*_ =  −0.83 and *r*_*p*_ =  −0.48, and between RSarea and SWt where *r*_*g*_ = 0.05 and *r*_*p*_ = 0.28. With the exception of RAvdiam and Xsings, the *r*_*g*_ and *r*_*p*_ between the other root traits and SWt were generally larger under HN compared to the corresponding *r*_*g*_ and *r*_*p*_ between root traits and SWt under LN (Fig. 2; Tables 2 and 3). The close similarities between *r*_*g*_ and *r*_*p*_ between within an N-management show that in our experimental growth environment, the *r*_*g*_ and *r*_*p*_ between the root traits and SWt were close approximations of each other within an N-management, likely due to low effects of the environment external to the growth facility on genotypes. Across the N-managements, the *r*_*g*_ between the root traits and SWt were greater than the corresponding *r*_*p*_, most likely due to the between N-management treatment noise confounding the phenotypic variance on *r*_*p*_. As mentioned earlier, here, *H* was less than a threshold we had set at 0.1; therefore, *H* values for Xsings between the N-managements and *r*_*g*_ associated with it were not included in the across-management output (Table 4).

**Table 3:** The genetic correlations (lower triangle) and phenotypic correlations (upper triangle) of eight root trait and one shoot trait under low N-management.

Traits	SWt	RWt	RLenght	RSarea	RAvdiam	RVol	Tips	Forks	Xsings
SWt		0.515	0.529	0.284	− 0.481	0.112 ns	0.586	0.472	0.728
RWt	0.521		0.880	0.849	0.104 ns	0.769	0.777	0.887	0.793
RLenght	0.539	0.929		0.869	− 0.034 ns	0.639	0.881	0.936	0.870
RSarea	0.047 ns	0.676	0.744		0.431	0.931	0.662	0.871	0.587
RAvdiam	− 0.827	− 0.553	− 0.139	0.533		0.690	− 0.238	0.054 ns	− 0.310
RVol	− 0.296	0.218	0.439	0.920	0.794		0.419	0.685	0.324
Tips	0.623	0.745	0.822	0.405	− 0.424	0.115 ns		0.837	0.875
Forks	0.465	0.986	0.916	0.788	− 0.004 ns	0.538	0.755		0.831
Xsings	0.890	0.871	0.833	0.357	− 0.452	0.052 ns	0.885	0.760	

SWt, shoot weight, RWt, root weight, RLength, root length, RSarea, root surface area, RAVdiam, root average diameter, RVol, root volume, Tips, number of root tips, Forks, number of forks, Xsings, number of crossings, ns, not significant at P ≤ 0.05; all others, significant at 0.05 > p < 0.001.

**Table 4:** The genetic correlations (lower triangle) and phenotypic correlations (upper triangle) between traits across the two N managements.

Traits	SWt	RWt	RLenght	RSarea	RAvdiam	RVol	Tips	Forks	Xsings
SWt		0.759	0.684	0.576	0.020 ns	0.406	0.618	0.640	0.692
RWt	0.952		0.878	0.843	0.194	0.692	0.760	0.861	0.817
RLenght	0.999	0.999		0.934	0.116 ns	0.755	0.903	0.957	0.929
RSarea	0.999	0.999	0.999		0.423	0.936	0.785	0.906	0.788
RAvdiam	0.999	0.999	0.999	0.999		0.664	− 0.099 ns	0.132 ns	− 0.038 ns
RVol	0.988	0.999	0.999	0.999	0.999		0.587	0.750	0.574
Tips	0.629	0.911	0.905	0.719	0.702	0.751		0.885	0.869
Forks	0.999	0.999	0.976	0.855	0.999	0.961	0.980		0.940
Xsings	NA	NA	NA	NA	NA	NA	NA	NA	

SWt, shoot weight, RWt, root weight, RLength, root length, RSarea, root surface area, RAVdiam, root average diameter, RVol, root volume, Tips, number of root tips, Forks, number of forks, Xsings, number of crossings, ns, not significant at P ≤ 0.05; all others, significant at 0.05 > p < 0.001, *NA* Not available.

### Variation among traits and between N managements

Variation among the genotypes and in the genotype × management had a significant effect for all the traits in the two N managements, but RAvdiam had the least variation among the genotypes with CV ~ 10.3% in the HN and ~ 7.1% in the LN, compared to CV ranging between ~ 47% to ~ 80% among the rest of the traits in the two N managements (Table 6). Because CV is highly dependent on the grand mean of a trial^50^, we exercised caution in using a CV to interpret the comparative variability between traits under the two N managements. The trait CV between the two N management did not show any specific pattern to suggest a trait variance inflation due to the low nitrogen treatment or the differences in the grand means.

### Comparing heritability and correlated response in LN and HN among root traits

The mean *H* of only two traits, SWt and Xsings, were substantially greater under HN than under LN, while the mean *H* of RAvdiam was substantially greater under LN than under HN. Trait heritability showed varying degrees of *H* ‘instability’ between the two N-managements (Table 5). Some showed higher *H* under one N management than the other, with RAvdiam being the most heritable (under LN) and having the largest *H* difference between the N-managements (53.6% in LN and 95.7% in LN). RWt had the least difference (50.3% in HN and 50.6% in the LN). *H* between the N-managements was very low, yet the genetic correlation was very high, suggesting that *H* is affected by the environmental noise between the two N-managements.

**Table 5:** Variance, heritability and means separation for shoot weight and root traits.

Statistic	N management	SWt	RWt	RLength	RSarea	RAdiam	RVol	Tips	Forks	Xsings
Heritability	HN	0.646	0.503	0.649	0.605	0.536	0.554	0.655	0.634	0.658
	LN	0.701	0.506	0.417	0.475	0.957	0.634	0.455	0.471	0.588
	Combined	0.118	0.165	0.081	0.205	0.0996	0.229	0.141	0.166	0.0415
Genotype Variance	HN	4.740***	0.073***	83,728.7***	476.8***	0.00023***	0.0179***	171,457.8***	2,050,402.7***	171,116.6***
	LN	0.430***	0.0404***	24,232.9***	341.8***	0.0039***	0.055***	115,541.5***	754,145.1***	35,187.6***
	Combined	0.265	0.0103	3942.1	86.02	0.000118	0.0078	19,511.98	218,626.9	3391.99
Management Variance	Combined	6.275***	0.020**	5767.0*	16.6	0.0035***	0.0155***	75.89	0	46,191.8***
Genotype × Management Variance	Combined	2.495***	0.048****	50,039.5***	323.3	0.0019***	0.029***	123,987.7***	1,179,157.3***	99,761.8***
Residual Variance	HN	7.791	0.215	135,956.9	934.52	0.0006	0.044	271,071.4	3,549,899	266,834.7
	LN	0.549	0.119	101,570.5	1132.45	0.0005	0.096	416,459.7	2,539,164	73,997.1
	Combined	4.461	0.171	118,741.2	1033.6	0.0006	0.0697	343,826.2	3,043,825	170,311.1
Trait Mean	HN	5.172	0.657	694.3	52.8	0.238	0.324	1106.1	2417.2	672.2
	LN	1.623	0.456	581.7	59.22	0.322	0.501	1157.7	2358.7	365.4
LSD on Mean	Combined	1.356*	0.261	167.9	23.08	0.0287*	0.216	361.1	1190.7	159.02*
CV	HN	53.97	70.61	53.11	57.9	10.281	64.44	47.07	77.947	76.85
	LN	45.65	75.54	54.79	56.82	7.133	61.83	55.74	67.56	74.44
	Combined	62.23	73.94	54.01	57.4	8.474	64.06	51.81	73.06	79.54

LN, low N; HN, high N; LSD, least significant difference; CV, coefficient of variations; *, **, ***significant at p = 0.05, 0.05 > p ≥ 0.01, p < 0.01, respectively.

RAvdiam had significant negative and large correlations (− 0.83) to SWt under LN, while the correlations were positive but not significant under HN. It was ranked among the highest in the RF regression under LN but ranked bottom in the HN. The heritability was very high (0.957) under LN compared to 0.536 under HN. On the other hand, Xsing, was ranked highest ranked by the RF regressions under LN, but lower under HN (*r*_*g*_ and *r*_*p*_ were positive and highly significant in both managements, while *H* was medium' in the LN, 0.588; in the HN, 0.658). These observations suggest that selecting for small root diameter may be desirable for improving shoot weight of baby spinach in low N. In fact, of all the root traits in this report, only RAvdiam had a predicted high indirect selection efficiency (113%) for SWt (r_g_RTswt_ × H_RT_ > H_swt_, Table 6). Stronger correlated response efficiency of SWt was predicted for morphological (RLength and RAvdiam) and architectural traits (Tips and Xsings) compared to the standing crop root traits (RSarea, RVol, and RWt) (Table 6).

**Table 6:** Predicting the correlated response and indirect selection efficiency for shoot weight using root traits as the secondary traits under high N-management and low N-management.

	H_swt_	V_g-swt_	Root trait	r_g_	H_trait_	CR_swt_	R_swt_	CR_swt_/R_swt_	Indirect selection efficiency
High N-management	0.646	4.740	RWt	0.804	0.504	1.243	1.750	0.710	0.627
			Rlenght	0.769	0.649	1.349		0.771	0.772
			RSarea	0.757	0.605	1.282		0.733	0.709
			Ravdiam	0.045	0.536	0.072		0.041	0.037
			RVol	0.730	0.554	1.183		0.676	0.626
			Tips	0.815	0.655	1.436		0.821	0.826
			Forks	0.703	0.634	1.219		0.696	0.690
			Xsings	0.706	0.658	1.247		0.713	0.719
Low N-management	0.701	0.430	RWt	0.521	0.506	0.243	0.549	0.443	0.376
			Rlenght	0.539	0.417	0.228		0.416	0.321
			RSarea	0.047	0.475	0.021		0.039	0.032
			Ravdiam	− 0.827	0.957	− 0.530		− 0.966	− 1.128
			RVol	− 0.296	0.634	− 0.155		− 0.281	− 0.268
			Tips	0.623	0.454	0.275		0.501	0.403
			Forks	0.465	0.472	0.209		0.381	0.313
			Xsings	0.890	0.588	0.447		0.815	0.746

V_*g-swt*,_ genetic variance of shoot weight (SWt), r_*g*_, genetic correlation between SWt and a root trait, H_*swt*,_ broad sense heritability (repeatability) of SWt on a line mean basis; H_*trait,*_ broad sense heritability (repeatability) a root trait on a line mean basis, CR_*swt*_, Correlated response; R_*swt*_, response to direct selection; Indirect selection efficiency compares *H*_*swt*_ to [(*r*_*g*_ between SWt and Trait) × *H*_*trait*_].

## Discussion

The analysis pipeline was designed to define the phenotypic, genotypic, and predictive relationship between root architecture traits (Forks, Tips, and Xsings), root morphological traits (RLength, and RAvdiam), the root system of the standing crop (RSarea, RVol, and RWt) and between the root traits and the SWt of spinach grown in a soilless system. The objective was to determine root traits that have the greatest effect on the harvestable shoot under low N and thus can be used as a secondary trait to select for high NUE germplasm. We determined the phenotypic and genetic correlations (*r*_*p*_ and *r*_*g*_, respectively) between root traits and between the root traits and the SWt within and between the N-managements. Parallel to the correlation analyses, we used the predictive random forest machine learning technique to rank the root phenotypes according to their strength to predict SWt.

### Selecting the root traits with predicted potential as secondary traits for shoot biomass

We predicted the correlated response (CR) in SWt resulting from selecting any of the eight root traits to speculate its suitability as a secondary trait. An important component in defining CR is the *H*, which integrates information on genetic variation and environmental noise into one statistic and thus is useful in planning breeding programs^51^. One condition that must be met for indirect selection to be effective is *H* and *r*_*g*_ must be high in both the selection and target environments^43,44^ even though *H* and *r*_*g*_ are environment- and population-specific^43,46^. Fortunately, *H* has been strikingly similar in many environments^34,52^, and *H* variations in indoor growth environments are expected to be low^52,53^. In this context, since our *H* estimates were the average repeatability of 3-replicate trials in each of the N-managements, it may also be used to estimate the correlation expected between line means obtained from trials conducted at different indoor systems. Selecting a root trait in one N-management where *H* is high may predict the performance in the other, but we think the actual phenotypic quantity may vary substantially*.* However, if heritability values are high for both traits, then the correlation in breeding values dominates the phenotypic correlation^43,45,46^. Since the *H* values in SWt (*H* ~ 70%) was not as high compared to *H* of RAvdiam (~ 96%), and with a genetic correlation of − 0.827 between them, the correlation in environmental values within N-management which dominated the phenotypic correlation (− 0.481) between RAdiam and SWt may have been mainly due to LN-management effect on SWt. Thus, an LN environment that minimizes the devaluation of the breeding values between the RAvdiam and SWt must be maintained, and we believe indoor environments may provide this condition.

In this context, selecting for a root trait as a secondary trait should produce a correlated response in spinach shoot biomass, and the ratio *CR*_*swt*_/*R*_*swt*_ (Table 5) provides such an indirect selection criterion. It is clear (from these ratios) that direct selection of shoot biomass (SWt) is predicted to be superior to selecting for most of the eight root traits as a proxy in both N-managements. The exception is RAvdiam, which was predicted to result in superior indirect selection efficiency (112.8%) for SWt, i.e., a gain of ~ 12.8% in SWt by selecting against large RAvdiam. Other traits resulted in lower than 100% predicted efficiency; for instance, under LN was Xsings (74.6%), while the rest were less than 45% efficient. In the HN management, RLength (77.2%) and Tips (82.6%) were the most efficient but not enough for an indirect selection advantage.

### The case for selecting against large average root diameter in baby spinach

We have noticed that under HN, RAvdiam did not have significant *r*_*g*_ to any of the other root traits and with SWt, and only had significant positive *r*_*p*_ with RWt and RVol (Table 2). Meanwhile, under LN, RAvdiam had significant positive *r*_*p*_ only to RSarea and RVol, non-significant *r*_*p*_ with RWt (0.104) and RLength, but significant negative *r*_*p*_ with Tips (− 0.238) and Xsings (− 0.310) (Table 3). Spinach requires high N supply^8^, and under such conditions, it seems RAdiam is not likely to substantially influence the yield differences observed in shoot biomass among the genotypes. However, the significant negative *r*_*p*_ (− 0.481) and the highly negative *r*_*g*_ (− 0.827) with SWt under LN management (Table 3) suggest that larger mean root diameter is associated with smaller mean shoot weight and vice versa. The reduction in root diameter-related phenes in the youngest maize nodes under N stress suggested that root diameter might play a role in adaptive stress responses^54,55^. Whether or not the greater mean RAvdiam was due to root girth expansion in a negative feedback response to low N or competing resource allocation^8,34^ was not investigated in this study. Based on the pattern of diameter change in response to nutrient concentration in different species, it is suggested that altering root diameter may be another way to save C costs in root growth during nutrient stresses^56^. Although it is unclear how the root anatomical changes influence spinach root diameter, maize roots showed reduced cell diameter and area of vessels but an increased amount of aerenchyma during LN stress^54^. It is plausible to assume that N is preferentially allocated to the roots to sustain their growth under LN than shoots, and the reduced N concentration act as the internal signal in regulating the response of axile root growth. Given the robust correlated response efficiency of SWt predicted for RAvdiam and lateral root traits (Tips and Xsings) in our study, the RAvdiam measure can be used to indicate the ratio of axial: lateral roots.

The genotypic correlations between RAvdiam and RSarea (0.533) and between RAvdiam and RVol (0.794) were also highly significant and positive. This implies that selecting against large root diameter may also select against RSarea and RVol under low N-management. Since there was non-significant *r*_*g*_ but significant *r*_*p*_ between RSarea and SWt, our data suggest that only a limited genetic linkage drag or pleiotropy^45^ on shoot yield might result from selecting against large RAvdiam. Moreover, RSarea and RVol are standing crop traits^37^ for ‘root bulk’, which are a product of morphological traits (RAvdiam and RLength) and root architectural traits (e.g., Tips, Forks, and Xsings). The significant small positive *r*_*p*_ between RSarea and SWt may be a combined artifact of these other morphological and architectural components when we also consider a significant negative *r*_*p*_ existed between RAvdiam and SWt in the LN management. This position is also supported by the fact that RVol had a negative *r*_*g*_ (− 0.296) and an insignificant *r*_*p*_ to SWt, and yet RVol had positive *r*_*g*_ (0.439) and *r*_*p*_ (0.931) to RLength; RLength, on the other hand, had significant *r*_*g*_ (0.539) and *r*_*p*_ (0.529) to SWt. RAvdiam was also highly heritable with *H* ~ 96% under LN, Table 6). We propose that the root average diameter is the only trait in this study that can successfully be selected against to improve the yield of shoot biomass low N. Further studies to validate these findings might benefit from testing in multiple growth conditions (temperature, humidity, and graduated N concentrations) to define a broader range of root trait-shoot yield relationships and N-responsiveness.

### Resolving the conundrum around the antagonistic relationship between RAvdiam and SWt

Compared to SWt, RAvdiam has shown greater *H* (Table 6)*.* The *r*_*g*_ between SWt and RAvdiam can be high (Table 3). In other words, indirect selection for a secondary trait will be superior if the heritability of that trait is high, and the correlation between the traits is close to 1^45,46^. In this study, the RAvdiam met these two critical criteria. However, for practical use in a breeding program, the secondary trait must also be inexpensive and easy to measure in large trials^43,45^. In that case, shoot biomass estimate could be used to select for roots bulk traits in production systems that target spinach roots as the end product. Because precision in imaging techniques for roots and shoot is rapidly evolving^57,58^, we believe that soilless systems can be designed to facilitate robust root metrics characterization to match the ease with which above-ground biomass can be phenotyped. It would also be worthwhile to determine if further selection for/ against other root traits would eventually result in a superior secondary selection for shoot biomass. How these relationships would play along as the plants mature under different indoor growth conditions or in the field conditions require further studies.

Although a soilless system reduces the complexities associated with soils, we believe that the selection of a root trait needs to be understood in the context of the possible complex interplay among root traits^45,51^. The possible complex interactions between the root traits that may have influenced the SWt were not explicitly considered in our interpretations. However, we have alluded to such complexity by describing the trait to trait correlations, which we hope should serve as an impetus for further inquiry. Although the expected genetic correlation between estimates of cultivar means are best obtained from independent sets of trials^43,59^, we hypothesize that under similar N treatments, manipulation of other growth conditions in independent indoor growth environments may lead to some deviations from the response to selection predicted here. With the advent of techniques in processing images and the deep learning^60^ frameworks that use advanced optimization and features from data, such prediction accuracy is likely to improve continually^61–63^. Nonetheless, machine learning recognized root traits would continue to rely on vigorous calibrations and field-based validation in systems of interest.

In conclusion, we report on the investigation of eight root traits genetic and phenotypic correlations with fresh shoot biomass of spinach grown in a soilless system in a controlled indoor environment. The plants were harvested at 41 d after sowing, a stage corresponding to the marketable baby spinach. We have used both genotypes by management and other conventional breeder statistics and a machine learning predictive technique to define candidate root traits with the potential for indirectly selecting for spinach shoot yield. The experiments were set up under two separate and contrasting N-managements. Of the eight root traits, the root average diameter emerged as the only candidate with a predicted indirect selection efficiency good enough to improve shoot biomass. However, it had a robust negative genetic correlation with shoot yield, making us believe that selecting against large root diameter may improve the fresh shoot yield of baby spinach. We have exercised caution in this interpretation by recommending further studies into the possible complex interactions among the root traits considered in improving shoot biomass yield in baby spinach.

## Supplementary Information

Supplementary Information.

## Data Availability

All data generated or analyzed during this study are included in this published article (and Supplementary Information files).

## References

[1] Gruber BD, Giehl RF, Friedel S, von Wirén N (2013). Plasticity of the Arabidopsis root system under nutrient deficiencies. Plant Physiol..

[2] Sun C-H, Yu J-Q, Hu D-G (2017). Nitrate: a crucial signal during lateral roots development. Front. Plant. Sci..

[3] Socolow RH (1999). Nitrogen management and the future of food: lessons from the management of energy and carbon. Proc. Natl. Acad. Sci..

[4] Marvi MSP (2009). Effect of nitrogen and phosphorous rates on fertilizer use efficiency in lettuce and spinach. J. Hortic. For..

[5] Schenk M, Heins B, Steingrobe B (1991). The significance of root development of spinach and kohlrabi for N fertilization. Plant Soil.

[6] Stagnari F, Di Bitetto V, Pisante M (2007). Effects of N fertilizers and rates on yield, safety and nutrients in processing spinach genotypes. Sci. Hortic..

[7] Biemond H, Vos J, Struik P (1996). Effects of nitrogen on accumulation and partitioning of dry matter and nitrogen of vegetables. 3. Spinach. NJAS Wageningen J. Life Sci..

[8] Smorlders E, Merckx R (1992). Growth and shoot:root partitioning of spinach plants as affected by nitrogen supply. Plant Cell Environ..

[9] Walch-Liu P, Forde BG (2008). Nitrate signalling mediated by the NRT1. 1 nitrate transporter antagonises l-glutamate-induced changes in root architecture. Plant J..

[10] Lima JE, Kojima S, Takahashi H, von Wirén N (2010). Ammonium triggers lateral root branching in Arabidopsis in an AMMONIUM TRANSPORTER1; 3-dependent manner. Plant Cell.

[11] Forde BG (2014). Nitrogen signalling pathways shaping root system architecture: An update. Curr. Opin. Plant Biol..

[12] Giehl RF, Gruber BD, von Wirén N (2014). It’s time to make changes: Modulation of root system architecture by nutrient signals. J. Exp. Bot..

[13] Razaq M, Zhang P, Shen H-L, Salahuddin A (2017). Influence of nitrogen and phosphorous on the growth and root morphology of Acer mono. PLOS ONE.

[14] Lee S, Lee J (2015). Beneficial bacteria and fungi in hydroponic systems: Types and characteristics of hydroponic food production methods. Sci. Hortic..

[15] Parniske M (2008). Arbuscular mycorrhiza: the mother of plant root endosymbioses. Nat. Rev. Microbiol..

[16] Eldridge BM (2020). Getting to the roots of aeroponic indoor farming. New Phytol..

[17] Liese R, Alings K, Meier IC (2017). Root branching is a leading root trait of the plant economics spectrum in temperate trees. Front. Plant. Sci..

[18] Gopinath P, Vethamoni I, Gomathi M (2017). Aeroponics soilless cultivation system for vegetable crops. Chem. Sci. Rev. Lett..

[19] Koohakan P (2004). Evaluation of the indigenous microorganisms in soilless culture: Occurrence and quantitative characteristics in the different growing systems. Sci. Hortic..

[20] Zhao J, Bodner G, Rewald B (2016). Phenotyping: Using machine learning for improved pairwise genotype classification based on root traits. Front. Plant Sci..

[21] Bodner G (2013). A statistical approach to root system classification. Front. Plant. Sci..

[22] Moon T, Ahn TI, Son JE (2018). Forecasting root-zone electrical conductivity of nutrient solutions in closed-loop soilless cultures via a recurrent neural network using environmental and cultivation information. Front. Plant. Sci..

[23] Lammerts van Bueren ET, Struik PC (2017). Diverse concepts of breeding for nitrogen use efficiency. A review. Agron. Sustain. Dev..

[24] Chan-Navarrete, R., Dolstra, O., van Kaauwen, M., van Bueren, E. T. L. & van der Linden, C. G. Genetic map construction and QTL analysis of nitrogen use efficiency in spinach (*Spinacia oleracea* L.). *Euphytica* **208**, 621–636 (2016).

[25] Chan-Navarrete, R., Kawai, A., Dolstra, O., van Bueren, E. T. L. & van der Linden, C. G. Genetic diversity for nitrogen use efficiency in spinach (*Spinacia oleracea* L.) cultivars using the Ingestad model on hydroponics. *Euphytica* **199**, 155–166 (2014).

[26] Ju C (2015). Root and shoot traits for rice varieties with higher grain yield and higher nitrogen use efficiency at lower nitrogen rates application. Field Crop Res..

[27] Mu X (2015). Genetic improvement of root growth increases maize yield via enhanced post-silking nitrogen uptake. Eur. J. Agron..

[28] SharathKumar M, Heuvelink E, Marcelis LFM (2020). Vertical farming: Moving from genetic to environmental modification. Trends Plant. Sci..

[29] Despommier D (2011). The vertical farm: Controlled environment agriculture carried out in tall buildings would create greater food safety and security for large urban populations. J. Verbr. Lebensm..

[30] Meinen E, Dueck T, Kempkes F, Stanghellini C (2018). Growing fresh food on future space missions: Environmental conditions and crop management. Sci. Hortic..

[31] Eppendorfer WH, Bille SW (1996). Free and Total Amino Acid Composition of Edible Parts of Beans, Kale, Spinach, Cauliflower and Potatoes as Influenced by Nitrogen Fertilisation and Phosphorus and Potassium Deficiency. J. Sci. Food Agric..

[32] Maneejantra N (2016). A quantitative analysis of nutrient requirements for hydroponics Spinach (*Spinacia oleracea* L.) production under artificial light in a plant factory. J. Fertil. Pest..

[33] Lynch J (1995). Root architecture and plant productivity. Plant. Physiol..

[34] Lynch, J. P. in *Nutrient Acquisition by Plants* Vol. 181 *Ecological Studies* (ed BassiriRad H.) Ch. Chapter 7, 147–183 (Springer, 2005).

[35] Wright MN, Zagger A (2017). ranger: A fast implementation of random forests for high dimensional data in C++ and R. J. Stat. Softw..

[36] Alvarado, G. *et al.* (eds Maize International & Center Wheat Improvement) (CIMMYT Research Data & Software Repository Network, 2015).

[37] Iversen, C. M., McCormack, M. L., Blackwood, C. B., Freschet, G. T., Kattge, J., Roumet, C., Stover, D. B., Soudzilovskaia, N.A., Valverde-Barrantes, O. J., van Bodegom, P. M., Violle, C. Version 2 (Department of Energy, Oak Ridge National Laboratory TES SFA, U.S., Oak Ridge, Tennessee, USA, 2018).

[38] Breiman, L. in *Manual On Setting Up, Using, And Understanding Random Forests V3.1 (University of California at Berkeley, Berkeley, CA)* (2002).

[39] Breiman L (2001). Random forests. Mach. Learn..

[40] Falconer, D. S., Mackay, T. F. & Frankham, R. Introduction to quantitative genetics (4th edn). *Trends in Genetics*, Vol. 12, p. 280 (1996).

[41] Cooper M, DeLacy I (1994). Relationships among analytical methods used to study genotypic variation and genotype-by-environment interaction in plant breeding multi-environment experiments. Theor. Appl. Genet..

[42] Ward JH (1963). Hierarchical grouping to optimize an objective function. J. Am. Stat. Assoc..

[43] Falconer, D. S. *Introduction to Quantitative Genetics*. 365 (Ronald Press, 1961).

[44] Searle SR (1965). The value of indirect selection: I. Mass selection. Biometrics.

[45] Gallais, A. in *Efficiency in Plant Breeding.* (ed W. Lange, Zeven, A.C., Hogenboom, N.G. ) 45–60 (Pudoc, 1984).

[46] Hansel, H. in *Efficiency in Plant Breeding.* (ed A.C. Zeven and N.G. Hogenboom W. Lange) 61–64 (Pudoc, 1984).

[47] Liaw A, Weggy M (2002). Classification and regression by randomForest. R News.

[48] Stekhoven DJ, Bühlmann P (2012). MissForest—non-parametric missing value imputation for mixed-type data. Bioinformatics.

[49] Ljumović, M. & Klar, M. in *2015 4th Mediterranean Conference on Embedded Computing (MECO).* 212–215 (IEEE).

[50] Brown, C. E. in *Applied multivariate statistics in geohydrology and related sciences* 155–157 (Springer, 1998).

[51] Wray NV (2008). P. Estimating trait heritability. Nat. Educ..

[52] Gitonga VW (2014). Genetic variation, heritability and genotype by environment interaction of morphological traits in a tetraploid rose population. BMC Genet.

[53] Folta KM (2019). Breeding new varieties for controlled environments. Plant. Biol..

[54] Gao K, Chen F, Yuan L, Zhang F, Mi G (2015). A comprehensive analysis of root morphological changes and nitrogen allocation in maize in response to low nitrogen stress. Plant. Cell Environ..

[55] Yang JT, Schneider HM, Brown KM, Lynch JP (2019). Genotypic variation and nitrogen stress effects on root anatomy in maize are node specific. J. Exp. Bot..

[56] Zobel RW, Kinraide TB, Baligar VC (2007). Fine root diameters can change in response to changes in nutrient concentrations. Plant. Soil.

[57] Bodner G, Nakhforoosh A, Arnold T, Leitner D (2018). Hyperspectral imaging: A novel approach for plant root phenotyping. Plant. Methods.

[58] Atkinson JA, Pound MP, Bennett MJ, Wells DM (2019). Uncovering the hidden half of plants using new advances in root phenotyping. Curr. Opin. Biotechnol..

[59] Holland, J. W., Nyquist, W.E., Cervantes-Martinez, T.C. in *Plant Breeding Reviews* Vol. 22 (ed J. Janick) Ch. 2, 29–39 (Wiley, 2003).

[60] LeCun Y, Bengio Y, Hinton G (2015). Deep learning. Nature.

[61] Khaki, S., Wang, L. & Archontoulis, S. *A CNN-RNN Framework for Crop Yield Prediction*. (2019). 10.3389/fpls.2019.01750 PMC6993602 32038699

[62] van Dijk ADJ, Kootstra G, Kruijer W, de Ridder D (2021). Machine learning in plant science and plant breeding. iScience.

[63] Shahhosseini M, Hu G, Huber I, Archontoulis SV (2021). Coupling machine learning and crop modeling improves crop yield prediction in the US Corn Belt. Sci. Rep..

