# Supplementary material for: Selection of nitrogen responsive root architectural traits in spinach using machine learning and genetic correlations
Source: Sci Rep. 2021 May 5;11:9536. doi: 10.1038/s41598-021-87870-z (PMC8100178; doi:10.1038/s41598-021-87870-z)
Supplement: Supplementary file 1 — Supplementary Information. [file 41598_2021_87870_MOESM1_ESM.pdf]

Title Page: Supplementary Dataset

Selection of Nitrogen Responsive Root Architectural Traits in Spinach Using Machine Learning and Genetic Correlations

Henry O. Awika, Amit K Mishra, Haramrit Gill, James DiPiazza, Carlos A. Avila, and Vijay Joshi*

* Corresponding author: Vijay.Joshi@tamu.edu

1. Supplementary Dataset Figure S1 - Root architecture images of two representative accessions under HN and LN.

2. Supplementary Dataset Table S1 - Composition of macro/micro-nutrients in the liquid media used for high and low N management

3. Supplementary Dataset Table S2 - Hypergrid search for model optimal tuning parameters

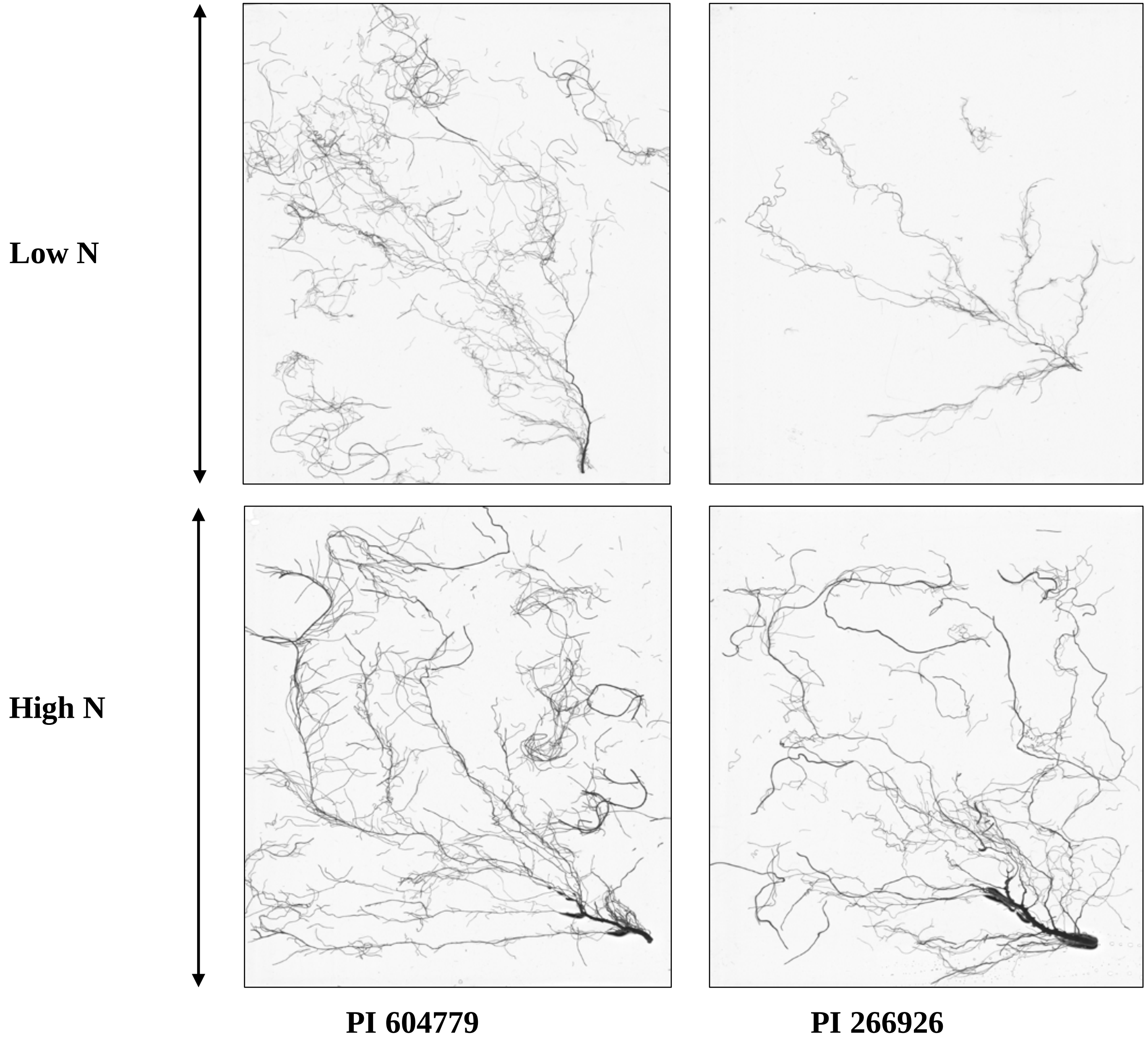

Supplementary Dataset Figure S1. Root architecture images of two representative accessions under HN and LN

Supplementary Dataset Table S1. Composition of macro/micro-nutrients in the liquid media used for high and low N management

	Concentration (ppm)	
**Elements**	**Low N**	**High N**
Nitrogen	50	150*
Phosphorous	48	48
Potassium	216	216
Magnesium	60	60
Sulfur	80	80
Iron	3	3
Manganese	0.5	0.5
Zinc	0.15	0.15
Copper	0.15	0.15
Boron	0.5	0.5
Molybdenum	0.1	0.1
Calcium	154.6	154.6

Supplementary Dataset Table S2. Hypergrid search for model optimal tuning parameters

RMSE, root mean square error

Model run ranking	High N-management				Low N-management			
	Random variable (mtry)	Node size	Sample size	Out-of-bag RMSE	Random variable (mtry)	Node size	Sample size	Out-of-bag RMSE
1	7	2	0.56	1.581197	3	8	0.7	0.4530081
2	6	8	0.56	1.584564	4	8	0.56	0.4538715
3	4	8	0.6	1.584709	5	8	0.56	0.4546246
4	6	4	0.74	1.584774	3	2	0.56	0.4549928
5	4	8	0.74	1.585096	3	8	0.6	0.4551021
6	8	2	0.74	1.585909	3	6	0.7	0.4551335
7	6	2	0.74	1.586207	4	8	0.632	0.4551744
8	7	8	0.56	1.586344	3	8	0.56	0.4552718
9	7	6	0.56	1.586575	4	6	0.56	0.4553085
10	6	8	0.74	1.586743	3	6	0.56	0.4553185
11	8	4	0.74	1.587177	4	6	0.632	0.4553972
12	7	6	0.74	1.587309	5	8	0.6	0.4554499
13	4	6	0.74	1.587499	2	8	0.6	0.4558534
14	6	4	0.7	1.587669	2	6	0.6	0.4558825
15	6	6	0.56	1.587776	2	8	0.74	0.4560114
16	8	8	0.6	1.587815	5	6	0.56	0.456166
17	4	6	0.632	1.588005	5	6	0.6	0.4562887
18	6	6	0.6	1.588282	4	6	0.6	0.4564574
19	6	8	0.632	1.588419	3	6	0.6	0.4565152
20	6	8	0.6	1.588743	5	8	0.632	0.4565187
21	4	6	0.6	1.588816	5	4	0.56	0.4565443
22	4	8	0.632	1.588958	3	8	0.74	0.4567529
23	7	4	0.56	1.589383	2	4	0.6	0.4568042
24	6	8	0.7	1.589393	3	4	0.56	0.4568766
25	7	8	0.74	1.589754	6	8	0.56	0.4571761
26	8	6	0.6	1.589868	4	4	0.56	0.457285
27	8	6	0.74	1.589975	4	8	0.6	0.4573764
28	8	8	0.74	1.590161	5	4	0.6	0.4574067
29	6	6	0.632	1.590355	7	8	0.6	0.45753
30	4	4	0.74	1.59041	5	2	0.56	0.4575688
31	5	8	0.7	1.59046	5	4	0.632	0.4576572
32	7	4	0.74	1.590529	4	4	0.632	0.457809
33	6	6	0.74	1.590934	5	6	0.632	0.4579287
34	6	6	0.7	1.590964	4	2	0.632	0.4580185
35	8	4	0.6	1.591142	6	8	0.6	0.4580591
36	6	2	0.56	1.591197	2	8	0.56	0.4580665
37	4	8	0.56	1.591292	4	8	0.7	0.4580909
38	4	4	0.6	1.591507	3	4	0.7	0.4582483
39	5	6	0.632	1.591747	2	4	0.74	0.4583056
40	6	4	0.56	1.591869	2	6	0.74	0.4585545
41	5	4	0.7	1.591873	2	8	0.8	0.4585695

42	5	2	0.7	1.591898	4	4	0.6	0.4586535
43	4	2	0.74	1.592176	2	8	0.7	0.4587368
44	4	2	0.6	1.592291	2	8	0.632	0.4588373
45	7	2	0.74	1.592392	4	8	0.8	0.4589037
46	6	4	0.6	1.592659	5	2	0.6	0.4589447
47	6	2	0.7	1.593308	2	6	0.56	0.4591657
48	7	8	0.6	1.593493	3	2	0.7	0.4591918
49	5	4	0.632	1.593561	3	6	0.74	0.4592017
50	5	8	0.632	1.593648	7	6	0.6	0.4592571
51	4	4	0.632	1.593754	3	4	0.74	0.4592704
52	8	8	0.56	1.593807	7	8	0.56	0.4593528
53	5	4	0.56	1.593931	4	2	0.56	0.4593691
54	4	4	0.56	1.593958	2	2	0.8	0.4593798
55	6	2	0.6	1.594246	5	8	0.7	0.4593921
56	8	2	0.6	1.594262	6	8	0.632	0.4595309
57	5	8	0.56	1.594702	3	4	0.6	0.4595446
58	5	2	0.56	1.59471	2	4	0.8	0.459545
59	5	6	0.7	1.595067	3	8	0.632	0.4595662
60	7	8	0.8	1.595303	2	6	0.632	0.4595799
61	7	2	0.6	1.595357	5	8	0.74	0.4596531
62	4	6	0.7	1.595745	3	4	0.632	0.4596945
63	8	6	0.56	1.595808	4	6	0.7	0.4597243
64	6	4	0.632	1.596114	3	2	0.6	0.4597527
65	8	8	0.7	1.596408	2	4	0.56	0.4598286
66	8	8	0.8	1.596564	2	2	0.74	0.4598763
67	5	8	0.6	1.596992	3	6	0.632	0.4598817
68	7	2	0.8	1.597019	2	6	0.8	0.4599668
69	8	4	0.56	1.597097	5	6	0.7	0.4600132
70	4	8	0.7	1.597382	4	2	0.7	0.4600522
71	5	8	0.74	1.597391	4	6	0.8	0.4601164
72	6	2	0.632	1.597667	6	6	0.6	0.4601429
73	5	4	0.74	1.597903	6	8	0.74	0.4601918
74	4	2	0.56	1.597997	6	6	0.56	0.4602032
75	7	6	0.6	1.598028	4	2	0.6	0.4602643
76	4	6	0.56	1.598081	4	8	0.74	0.4604018
77	4	2	0.632	1.598178	2	4	0.632	0.4605227
78	5	6	0.56	1.598279	2	6	0.7	0.4606215
79	5	6	0.74	1.598286	3	2	0.632	0.4606895
80	6	8	0.8	1.598888	5	2	0.632	0.4607219
81	7	8	0.632	1.598952	7	4	0.6	0.4607898
82	3	2	0.7	1.59973	2	2	0.6	0.4608603
83	7	6	0.8	1.599845	8	8	0.6	0.4610058
84	7	8	0.7	1.600145	4	4	0.8	0.4610321
85	7	4	0.6	1.600216	4	2	0.8	0.4610753
86	3	8	0.632	1.600379	3	2	0.74	0.461109
87	8	2	0.8	1.6004	6	2	0.6	0.4611332
88	3	4	0.632	1.60057	6	4	0.6	0.4611471

89	7	4	0.632	1.600983	6	4	0.632	0.4611668
90	8	2	0.56	1.601021	1	2	0.7	0.4613017
91	3	4	0.8	1.601243	2	4	0.7	0.4613563
92	3	8	0.8	1.601274	8	4	0.6	0.4613733
93	3	4	0.7	1.601379	8	8	0.56	0.4613947
94	3	8	0.7	1.601652	6	6	0.632	0.4614089
95	8	6	0.7	1.601658	4	4	0.7	0.4616119
96	5	6	0.8	1.601969	3	8	0.8	0.4617492
97	3	6	0.632	1.601998	5	6	0.74	0.4618521
98	7	6	0.7	1.602487	6	4	0.56	0.4618771
99	8	8	0.632	1.602528	7	8	0.632	0.4619875
10	3	8	0.6	1.602663	8	6	0.6	0.4620164
10	7	4	0.8	1.602707	7	6	0.56	0.4620418
10	2	2	0.632	1.602731	6	2	0.632	0.4620803
10	6	2	0.8	1.602754	1	4	0.7	0.4620803
10	7	6	0.632	1.602777	5	2	0.8	0.4622296
10	5	8	0.8	1.602915	5	4	0.74	0.462263
10	3	2	0.632	1.603162	5	8	0.8	0.4623017
10	8	2	0.7	1.603206	2	2	0.56	0.4623864
10	7	2	0.632	1.603404	6	8	0.8	0.4624285
10	8	4	0.7	1.603444	6	4	0.74	0.4625014
11	3	6	0.8	1.60349	7	2	0.6	0.4625104
11	8	6	0.8	1.60371	5	2	0.7	0.4625456
11	7	4	0.7	1.604677	1	6	0.8	0.4626498
11	6	6	0.8	1.604754	2	2	0.7	0.4627398
11	8	4	0.8	1.604874	1	2	0.56	0.4628672
11	5	2	0.632	1.604913	5	2	0.74	0.4628724
11	5	6	0.6	1.605104	1	6	0.74	0.4629028
11	4	4	0.7	1.60527	5	4	0.7	0.4630529
11	2	4	0.74	1.605655	7	4	0.74	0.4630913
11	5	2	0.74	1.605915	5	4	0.8	0.4632525
12	4	6	0.8	1.606108	4	6	0.74	0.4633627
12	5	2	0.6	1.606163	1	6	0.7	0.4634636
12	5	4	0.6	1.606487	6	6	0.74	0.4634883
12	4	2	0.7	1.606771	1	4	0.6	0.4635381
12	4	8	0.8	1.607085	7	4	0.56	0.4635518
12	7	2	0.7	1.607087	7	8	0.74	0.463585
12	3	8	0.56	1.607147	1	6	0.56	0.4636995
12	3	6	0.7	1.607486	4	2	0.74	0.4637673
12	2	4	0.632	1.607508	1	8	0.8	0.4638728
12	3	2	0.6	1.607768	2	2	0.632	0.4639202
13	2	6	0.74	1.607941	5	6	0.8	0.4639255
13	3	8	0.74	1.608016	3	6	0.8	0.4639451
13	2	6	0.632	1.608048	7	6	0.632	0.4639546
13	8	6	0.632	1.608077	6	2	0.56	0.4639866
13	3	4	0.6	1.608296	8	2	0.6	0.464122
13	3	6	0.74	1.608462	1	4	0.74	0.464132

13	3	6	0.6	1.609001	7	8	0.7	0.4641493
13	2	2	0.8	1.609396	6	6	0.8	0.4641771
13	5	4	0.8	1.60954	1	4	0.56	0.464423
13	3	2	0.8	1.609757	7	2	0.74	0.4644357
14	2	8	0.74	1.609851	8	6	0.56	0.464541
14	6	4	0.8	1.610733	1	8	0.74	0.4646062
14	8	2	0.632	1.610751	1	8	0.56	0.4646199
14	3	2	0.56	1.610993	6	8	0.7	0.4647266
14	2	6	0.8	1.611077	4	4	0.74	0.4648443
14	8	4	0.632	1.611766	6	6	0.7	0.4648812
14	2	8	0.632	1.611945	1	8	0.7	0.46491
14	5	2	0.8	1.612467	7	6	0.74	0.4650092
14	2	4	0.8	1.613216	7	4	0.632	0.4650856
14	3	4	0.56	1.613322	7	2	0.56	0.4652422
15	2	8	0.8	1.613419	7	2	0.632	0.4653297
15	3	4	0.74	1.613696	8	4	0.56	0.4653636
15	3	6	0.56	1.613814	1	6	0.6	0.4653904
15	2	8	0.7	1.614057	7	6	0.7	0.4653997
15	2	8	0.6	1.614452	3	4	0.8	0.4654424
15	4	4	0.8	1.614626	8	8	0.74	0.4656634
15	2	6	0.7	1.616684	8	8	0.632	0.4656999
15	2	6	0.56	1.617045	6	2	0.8	0.4659011
15	2	8	0.56	1.617535	1	2	0.6	0.4664377
15	2	2	0.74	1.617736	6	2	0.74	0.4664955
16	4	2	0.8	1.619445	6	4	0.8	0.4665205
16	2	6	0.6	1.619713	7	4	0.7	0.4666076
16	2	4	0.7	1.620262	6	4	0.7	0.4666866
16	2	2	0.7	1.620593	6	2	0.7	0.466721
16	3	2	0.74	1.620788	3	2	0.8	0.4668457
16	2	4	0.56	1.623486	1	4	0.8	0.4668769
16	1	8	0.632	1.625895	8	2	0.56	0.4671903
16	2	4	0.6	1.626981	8	4	0.632	0.4671927
16	2	2	0.56	1.627241	7	2	0.7	0.4672489
16	2	2	0.6	1.627255	8	6	0.632	0.4672946
17	1	6	0.632	1.63119	8	6	0.74	0.4673562
17	1	8	0.56	1.634972	1	6	0.632	0.467461
17	1	2	0.632	1.635213	8	8	0.7	0.4675083
17	1	4	0.56	1.63584	1	8	0.632	0.4675741
17	1	2	0.56	1.636765	8	6	0.7	0.4676865
17	1	2	0.6	1.637444	1	2	0.74	0.4677404
17	1	8	0.74	1.638064	1	4	0.632	0.4678951
17	1	6	0.74	1.640938	7	8	0.8	0.4679464
17	1	6	0.56	1.641906	8	4	0.7	0.4682727
17	1	8	0.6	1.643355	1	8	0.6	0.4683565
18	1	4	0.632	1.643401	8	4	0.74	0.4685208
18	1	6	0.6	1.644128	8	2	0.632	0.4691152
18	1	4	0.6	1.644448	7	6	0.8	0.4693086

18	1	8	0.7	1.644981	1	2	0.632	0.4695981
18	1	2	0.8	1.646379	7	4	0.8	0.4696445
18	1	6	0.8	1.646809	8	2	0.7	0.4699065
18	1	4	0.7	1.647681	8	2	0.74	0.4699478
18	1	2	0.7	1.64885	1	2	0.8	0.4703107
18	1	4	0.74	1.649542	7	2	0.8	0.4706115
18	1	6	0.7	1.650234	8	8	0.8	0.4713989
19	1	8	0.8	1.651234	8	6	0.8	0.4721185
19	1	4	0.8	1.652487	8	4	0.8	0.4738769
19	1	2	0.74	1.654104	8	2	0.8	0.4751906
